# How Preparation Protocols Control the Rheology of Organoclay Gels

**DOI:** 10.1021/acs.iecr.4c04467

**Published:** 2025-03-22

**Authors:** Nikolaos A. Burger, Benoit Loppinet, Andrew Clarke, George Petekidis

**Affiliations:** † IESL-FORTH, Vassilika Vouton, Heraklion 70013, Greece; ‡ Department of Materials Science & Engineering, University of Crete, Heraklion 70013, Greece; § SLB Cambridge Research, High Cross, Madingley Road, Cambridge CB3 0EL, U.K.

## Abstract

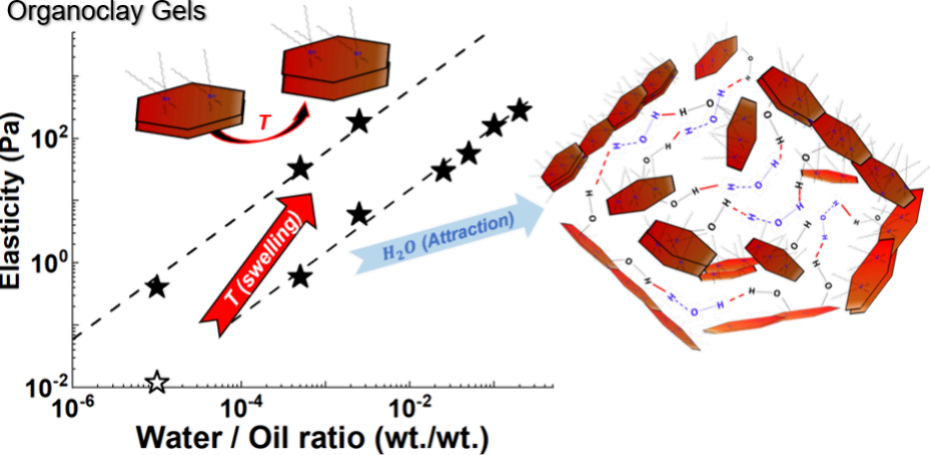

We elucidate the effect of preparation conditions on the rheological properties of organophilic clays consisting of platelet-like primary particles, VG69 (trademark of SLB) dispersed in oil, by varying the homogenization rate, homogenization temperature, and amount of added water. We establish that stable, nonsedimenting gel formation requires homogenization temperatures higher than 45 °C and the addition of a small amount of water during the homogenization stage. Dried organoclay dispersions, on the other hand, do not form stable gels, independent of the homogenization rate and temperature, suggesting the existence of only weak attractions in the absence of water molecules. Water-induced attraction is necessary to form gels, probably through hydrogen bonding between the silanol group of clay particles and water molecules. Moreover, the effect of homogenization temperature is related to the extent of exfoliation during the homogenization stage as confirmed by X-ray scattering. The gel plateau modulus, *G*_p_, is found to increase with clay concentration as *G*_P_ ∼ *c*_clay_^3.9^, typical of fractal gel networks. More interestingly, a linear increase in the elastic modulus with water concentration is observed over a wide range of water concentrations, while analyzing the effective yield strain deduced from the yield stress and elastic modulus reveals the existence of three regimes. We finally present dynamic state diagrams that clearly indicate the required conditions for the creation of stable gels and demonstrate the importance of controlling the preparation protocols in the formulation of clay dispersions and gels with desirable structural and mechanical properties.

## 1 Introduction

Organoclays are made up of clay particles modified by the adsorption of surfactants to turn their surface from hydrophilic to hydrophobic and enhance their compatibility with nonpolar matrices like organic solvents or polymer melts.^1−6^ Organoclays are used in a broad range of applications where they are typically dispersed in polymer or oil apolar matrices. Examples are enhancement of barrier properties of composite materials (for gases or liquids) used in the packaging industry,^7,8^ improvement of high-temperature material stability,^9^ as well as in adsorption compounds, paints, coatings, drilling fluids, polymer composites, cosmetics, etc.^7−22^

The rheology of organoclay dispersions in apolar solvents has been of interest for many years^11,23^ as understanding and controlling it^8,24,25^ represents a crucial step in optimizing their use in specific applications.^19,26−28^

Organoclay dispersions may form gels with moderate yield stress (Pa), an essential property for certain applications, in particular for drilling fluids.^19^ They may also exhibit shear-thinning, where viscosity decreases with increasing shear rate due to shear-induced structural changes, but also thixotropic response, where the viscosity and microstructure change rather slowly with time upon shear rate changes.^19,29−31^ Similar to other colloidal gels, shear history can also affect the rheological properties of organoclay gels, in a rather permanent (or very long-lived) way.^29^ Moreover, preparation parameters such as homogenization rate and temperature also influenced the final properties.^32^ Clearly, the parameters playing a role in setting the rheological properties are many, from the interparticle interactions and microscopic structures of the organoclay particles to the processing conditions. The shape of clay particles, the aspect ratio, the type and the concentration of surfactant as well as the polarity of the solvent are factors that matter.^11,33−39^ Well-selected preparation conditions can provide enhanced stability against sedimentation, increased modulus, and yield stress.^24,30,31,37,40,41^ Here we focus on the role of the preparation steps. In the typical dispersion process known as swelling and exfoliation,^32,42,43^ the organoclay in the powder form is mixed with a solvent (or polymer matrix), using mechanical agitation, sonication, and increased temperature to achieve a uniform dispersion^44^ in the solvent intended for the specific application.^45^ The process may induce further swelling and exfoliation of the not-fully exfoliated organoclay powder and affect the final properties of the dispersion. Further addition of surfactants or dispersants is sometimes used to achieve a stable dispersion and prevent reagglomeration of clay particles.^2,46^

An often neglected though relevant parameter is the possible presence of a small number of polar “contaminants” (e.g., water) in the apolar dispersion. The effect of the presence of polar molecules in the apolar medium is relevant in many types of systems with implications to the interaction balance and possible consequences on the rheology, from intercalation of polar molecules in the supramolecular aggregation of organo-gelators^47−49^ to the occurrence of capillary bridges when a large enough amount of a polar fluid is introduced in oil-based colloidal dispersions.^50−55^ In the case of organoclay dispersions, the effect of water presence on the rheology of dispersions has been noticed for many years.^4,42^ In particular, the addition of polar components is an old traditional engineering trick known as a ‘clay activation’. As is well established, small amounts of water (below percent) can significantly alter the rheology of the dispersions by the creation of stable, nonselective gels rather than fast sedimenting sol if no water is added. However, the role of the microscopic interactions between the polar solvent and the organoclay and its influence on the dispersion macroscopic mechanical properties remain to be fully elucidated.

In the following, we report a study using rheology of the effect of the preparation conditions on the macroscopic properties of the dispersions of organo-bentonite clays used in oilfield drilling fluid formulations.^26^ We investigated the effect of H_2_O concentration and homogenization temperature on both the linear and nonlinear rheological properties, in particular, the elastic plateau modulus the yield stress *G*_P_, σ_y_, and the shear rate-dependent viscosity η. The article is organized as follows: after details on methods and materials, we provide a characterization of the starting material comparing the organo-bentonite to the bentonite. We then introduce the different preparation protocols used and present their impact on the dispersion stability. Given the observed variation in stability, we provide X-ray scattering evidence of exfoliation during the preparation process. We then discuss in detail the rheological properties of plateau modulus and flow behavior and propose a tentative state diagram. The main findings are that the preparation steps are essential in controlling the mechanical properties with two main parameters besides the mixing energy, the temperature of the dispersion during the homogenization, and the water content.

## 2 Materials and Methods

### 2.1 Organoclay and Mineral Oil

We used commercial hydrophobically modified clays, VG69 (trademark of SLB) which is an amine-treated bentonite consisting of platelet-like particles.^42,56−58^ It is a frequently used viscosifying component of drilling fluid formulations in the oil and gas industry.^16,27^ Organoclays were dispersed in a drilling fluid base oil (Clairsol 370, Haltermann Carless) consisting of a mixture of hydrocarbons with a low (<1%) amount of aromatics with a high boiling point mineral oil (*T* > 280 °C). All chemicals were supplied by SLB.^19,26^

### 2.2 Dispersion Preparation Protocols

Dispersions were prepared using the hydrophobically modified organoclay and oil as received. As explained in more detail in the result part, we used different dispersion protocols, varying the rate of mixing, the homogenization temperature, and the amount of added water in order to investigate their influence on the structure and the mechanical properties of the final dispersions.

### 2.3 Experimental Procedure

Scanning electron microscopy (SEM) imaging was performed with a JEOL JSM 6390LV scanning electron microscope operated at 15–20 kV. Before sample imaging, all samples were dried and sputter-coated with ca. 10 nm of gold (Au). For shear rheology measurements, we used two stress-controlled rheometers (MCR-501 and MCR-302, Anton-Paar), operating in both stress- and strain-controlled modes. We utilized a homemade sandblasted cone plate (*d* = 40 mm, cone angle = 1.02 °, and truncation = 0.072 mm) and a homemade cover to suppress solvent evaporation, even though the Clairsol 370 oil has a high boiling point. The samples were always loaded and measured at 25 °C with the temperature regulated by a Peltier unit (±0.1 °C). To avoid slip effects, we also used a rough bottom plastic plate. Thermogravimetric analyzer (TGA) thermographs were obtained using an SDT600 TGA/DTA of TA Instruments under argon flow between 25 and 600 °C with a heating rate of 10 °C min^–1^. X-ray scattering, wide angle (WAXS), and small angle (SAXS) measurements were performed on a Xeuss 3.0 system (Xenocs) where the 2D X-ray scattering profiles were always isotropic and thus were azimuthally integrated to 1D data using Xenocs software.

For the high-water content VG69 dispersions, (*c*_H_2_O_ = 50 wt %) we used rheo-confocal in order to follow the structural evolution upon shear flow.^59^ For imaging, a confocal microscope (VT-Eye, Visitech International) was attached to the rheometer. A steel bottom plate with a rough glass coverslip (thickness = 170 μm) glued on top and a Nikon oil immersed 100× objective (NA = 1.45, working distance = 130 μm) was used. Also, a white light source was mounted next to the CCD camera for better illumination.

### 2.4 Rheological Protocol

To produce consistent and reproducible rheological data for such complex systems, rheological measurements need to be performed following strict protocols. Thus, we adopted the following shear protocol: after loading the samples into the rheometers we perform: (i) a preshear at 1000 s^–1^, for 60 s which is followed by a resting period of *t*_w_ = 1200 s. During this waiting period the evolution of *G*′, *G*″ is monitored by small amplitude oscillatory shear (SAOS) measurements at ω = 1 rad/s and γ = 0.1%. After the waiting period, a dynamic frequency sweep (DFS) at 0.01 ≤ ω ≤ 100 rad/s was performed to probe the linear viscoelasticity of the system.^29^ All further rheological tests were performed following the above protocol.

## 3 Results and Discussion

### 3.1 Organoclay Powder

**Figure 1:**
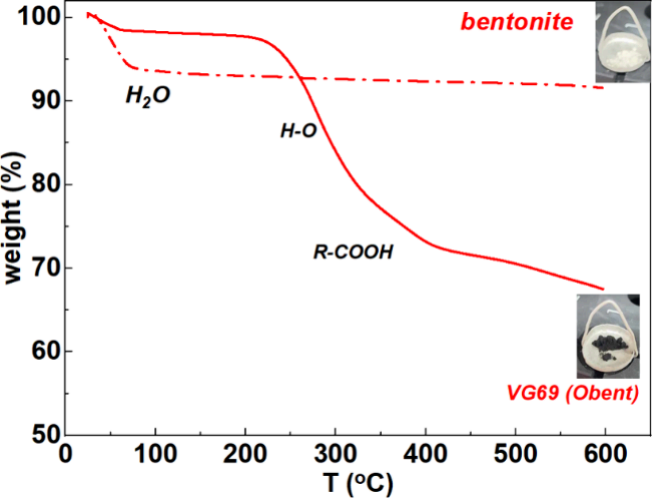
TGA thermographs of organoclay, organo-bentonite VG69 (red line), and natural bentonite (red dashed line) powders.

The extent of organic modification and the residual presence of water must be accounted for when considering the macroscopic properties of organoclay dispersions. We used Thermal Gravimetry Analysis (TGA) to quantify the amount of water and surfactant present in the organoclay and in the unmodified bentonite for comparison. The TGA thermographs of bentonite and organo-bentonite are plotted in Figure 1. In both compounds, we observe a low-temperature drop of mass, between 40 and 70 °C. This is attributed to the evaporation of both the “free” water molecules present in the powder (possibly between clay aggregates) as well as the water molecules bonded to the silanol groups of the clay particles via H-bonds.^1,42,60,61^ The corresponding water concentration, c_H_2_O_, is estimated to be 1 wt % in the organoclay powder and 7 wt % in the unmodified clay. The factor of 7 between the two corresponds to the expected increase of hydrophobicity after the clay surface modification with the quaternary ammonium surfactant.^1^ It shows the effectiveness of the surfactant to stop water adsorption. It is usually considered that the availability of water adsorption sites is decreased by at least 50 times upon the addition of the surfactants.^62^

The amount of water present can be used for an estimate of the level of microscopic water adsorption on the clay surface. Assuming a 100 m^2^/g surface per weight, typical for bentonite, 1 wt % water in the organoclay suspension corresponds to 10 mg of water per 100 m^2^ of clay surface or . Assuming liquid water type of density (*d* = 1 mg/mm^3^), it converts to the volume of water per surface of clay of , which amounts to a nominal thickness of 0.1 nm. This is less than the water molecular size so the water is not in a large enough amount to fully cover all the clay with a liquid-like layer but only a fraction of it. The 7% of water in the bentonite corresponds to 0.7 nm thickness, on the order of two molecular water thicknesses. In this case, liquid-like water could be covering the full clay surface.

The extra two-step decay function between 200 and 400 °C observed in the organoclay thermogram and not present in unmodified clay is attributed to the decomposition of hydroxyl and carboxyl groups in the surfactant. Based on the corresponding mass loss, the surfactant concentration is estimated to be *c*_surfactant_ = 30 wt %, well in excess compared to the available space in the clay surface or for intercalation between the clay particles.^1^

With respect to size characterization, we used SEM to confirm the clay particle shape and polydispersity and assess the presence of big aggregates in the clay powders. Typical pictures are shown in Figure S1. We should note that the large aggregates visible in the low-magnification pictures are expected to breakdown further and possibly exfoliate during the homogenization step of the dispersion preparation.

### 3.2 Different Dispersion Protocols and Resulting Properties

#### 3.2.1 Standard Protocol

**Figure 2:**
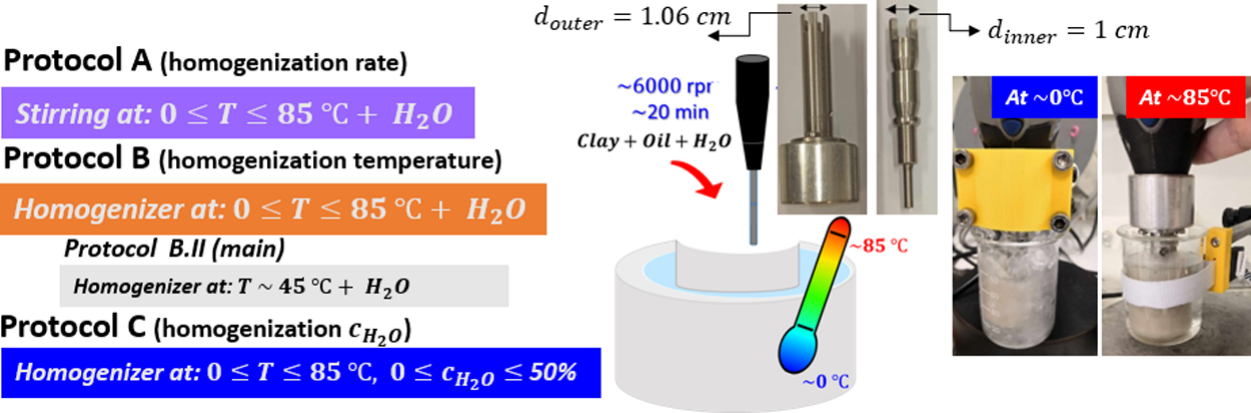
Cartoon shows from the left to right the three different protocols performed in this study, setup of sample preparation (not to scale): the sample vial is located in a water bath where the temperature is controlled via a thermostat in the temperature range 0 < *T* < 85 °C and shear is generated with a homogenizer at 6000 rpm for 20 min. The images show the inner and outer tools (with diameter values indicated) of the homogenizer. Photographs of sample preparation in an ice bath and at 85 °C in a water bath (for dry conditions, we used an oil bath instead of water).

The organoclay powder was gradually added to the mineral oil with the homogenizer turned on in order to avoid the formation of lumps. After a period of 10 min under mixing, Milli-Q water was added to reach a concentration of *c*_H_2_O_ = 2.5 × 10^–3^ wt/wt (or equivalent 0.25 wt %). Mixing was continued for 10 more minutes. Samples were then placed overnight on a bottle roller to ensure dispersion without air bubbles, while no solvent evaporation was detected during the preparation period. As we will discuss below, the properties of the dispersion are sensitive to the specific homogenization parameters, i.e., the homogenization rate, temperature, and *c*_H_2_O_. We define protocols in an effort to evaluate the effect of these parameters (Figure 2) as follows.

##### 3.2.1.1 Low Homogenization Rate (Protocol A)

A laboratory magnetic stirrer was used to prepare the dispersions instead of the high-shear homogenizer. The stirring time was extended to 2 days. The homogenization/dispersion temperature was varied between low and high temperatures (0, 25, or 85 °C) and the water concentration was kept at *c*_H_2_O_ = 0.25 wt %

##### 3.2.1.2 Varying Homogenization Temperature (Protocol B)

The homogenizer was used at 6000 rpm, producing a maximum shear rate, γ̇ = 2.1 × 10^4^ s^–1^, for at least 20 min.

When a 20 mL vial was placed in air (at 20 °C), the dispersion temperature during the high-shear mixing was measured to rise up to a steady temperature of 45 °C within a few minutes.

To allow better control of the dispersion temperature during homogenization, the 20 mL vial was placed in a water bath at controlled temperatures of ∼0 °C (contained in an ice bath to avoid heating to higher temperatures) (black symbols), 45 °C (red), 65 °C (olive), and 85 °C (magenta). The temperature range was chosen to be commensurate with working conditions of industrial drilling fluids, where a typical range is between 4 °C, (at sea floor offshore) and 65 °C. Although for geothermal wells temperatures typically reach up to 200 °C.^19,63^

##### 3.2.1.3 Varying Water Concentration, *c*_H_2_O_ (Protocol C)

Preweighted amounts of clay powders were dried in a vacuum oven at 60 °C for 24 h. Heating was stopped, and the temperature was allowed to reach room temperature before the pressure was increased back to atmospheric pressure by the introduction of air. The homogenizer was used at 6000 rpm for at least 20 min as above at homogenization temperature 0, 45, or 85 °C. *c*_H_2_O_ was adjusted through the addition of water covering a range from dry conditions (no added water) to 50 wt % H_2_O to oil ratio.

Visual inspection after preparation verified the good dispersion and the absence of sedimentation. Since organoclay particles are large and dense enough to induce clear sedimentation, their absence indicated the formation of a gel that can sustain its own weight and preclude sedimentation. We observed that a high homogenization rate was necessary for the formation of stable (nonsedimenting) organoclay. On the contrary, the absence of a strong homogenization rate (protocol A) always leads to fast (in a few minutes) sedimenting sol independent of the homogenization temperature, *c*_clay_ or *c*_H_2_O_.

**Figure 3:**
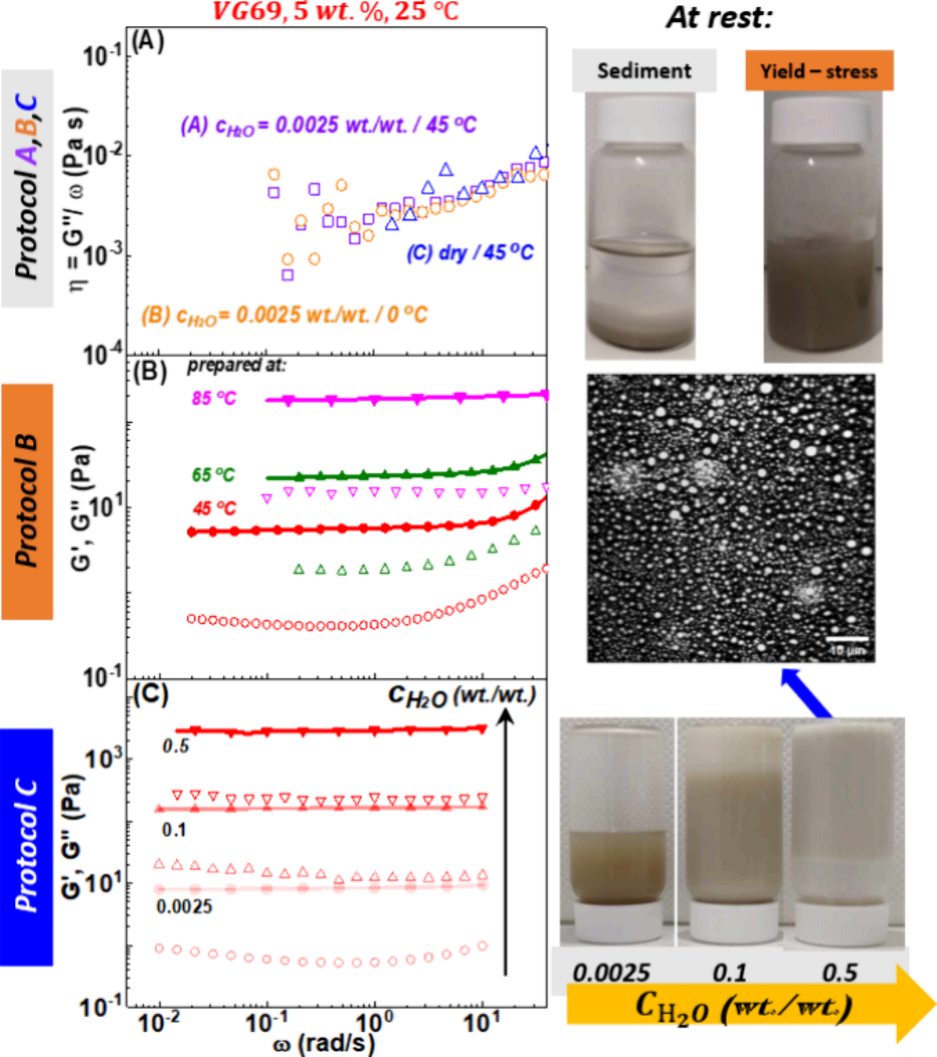
(A) Dynamic viscosity as a function of frequency of VG69 dispersions in Clairsol 370 prepared according to protocol A (purple squares), protocol B (orange circles), and protocol C (blue triangles). Storage (*G*″, full lines) and loss (*G*″, dashed lines) modulus (Pa) as a function of frequency of VG69 dispersions prepared according to (B) protocol B, at different homogenization temperatures at 45 (red), 65 (green) and 85 °C (magenta) at constant *c*_H_2_O_ = 0.25 wt % and (C) protocol C, samples prepared at 45 °C at different *c*_H_2_O_. Dynamic frequency sweep measurements are performed from high to low frequencies in the linear regime at γ = 0.1%, 25 °C. Right pictures: gel volume tests (after 1 h) at rest for dispersions prepared according to protocol A, B, and C, respectively. At larger c_H_2_O_, the gel does not move when the bottle is turned over, unlike the lower *c*_H_2_O_ gels.

The dispersions were further characterized by using linear rheology. Typical frequency-dependent storage (*G*′) and loss (*G*′) modulus of VG69 dispersions (*c* = 5 wt %) prepared according to protocols A, B, and C and measured at 25 °C are shown in Figure 3A–C.

Figure 3A illustrates the mechanical response of the nongelling dispersions, showing the frequency dependence of the viscosity, η = *G*″/ω. The dispersions remained liquid with measured viscosity of the order of a few mPa s, close to solvent viscosity (η_clairsol_ = 3.6 mPa s, at 25 °C). These liquids undergo a fast sedimentation, and we refer to them as unstable sols. They can be obtained under different conditions with all three of the preparation protocols. First, in all cases of low stirring rate (i.e., following protocol A) even in the presence of water. Second, at high homogenization rates (protocol B) and low homogenization temperatures, 0 °C, as well as at high homogenization rates and 45 °C (protocol C) in the absence of added water (dry conditions).

The other preparation protocols led to gelled samples with a clear frequency-independent plateau of the elastic modulus, *G*′, measured under SAOS. Figure 3B shows the frequency-dependent *G*′, *G*″ for dispersions prepared at different homogenization temperatures and constant homogenization rate with the water concentration at *c*_H_2_O_ = 0.25 wt % (protocol B). For preparation temperatures at 45, 65, and 85 °C, all samples exhibit a solid-like response (*G*′ > *G*″) with *G*′ almost frequency independent and tan(δ) 0.1 over the whole frequency range probed. These solid-like gel dispersions, resisted sedimentation for long periods of time, (∼months). Figure 3C shows the frequency dependence of *G*′, *G*″ at different *c*_H_2_O_ and constant homogenization rate and temperature (45 °C). We find that water content has a major influence on the viscoelasticity of the dispersions, and in particular, we observe that the plateau storage modulus (at ω = 1 rad/s), *G*_P_, increases gradually with *c*_H_2_O_. We further discuss this dependence in the next section.

The images shown on the right side of Figure 3 visually illustrate the different aspects and mechanical responses of the various organoclay dispersions. A typical sedimented unstable sol is shown in the top picture together with a greenish stable gel from protocol B. The bottom pictures illustrate the evolution from greenish to whitish with increasing water content. For *c*_H_2_O_ > 1 wt % the samples started to become whiter as a consequence of the presence of sizable water droplets (that increase light scattering). The sample with the larger water amount (*c*_H_2_O_ = 50 wt %) has the aspect of emulsions^64^ and is also seen (in the pictures at the bottom) to remain “stuck” in the vial when this is turned upside-down. A microscopy picture is shown in the middle row. The emulsions remain “stable” at rest for months up to high-water content (*c*_H_2_O_ ∼ 50 wt %). At the higher *c*_H_2_O_, the dispersion becomes very brittle and easily phase separated macroscopically into water-rich millimeter-size droplets and an oil-rich continuous phase (see Figure S5B). We refer to them as unstable emulsions.

**Figure 4:**
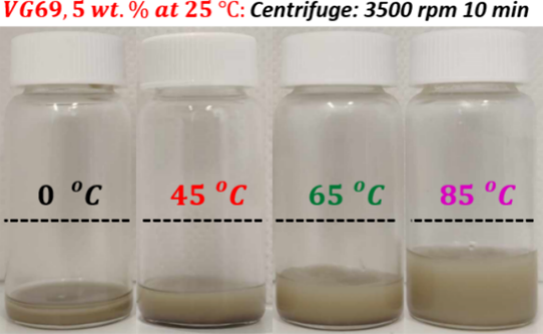
Gel volume experiments at constant VG69 concentration (5 wt %) performed at 25 °C for dispersions prepared according to protocol B at different homogenization temperatures and constant *c*_H_2_O_ = 0.25 wt %. after centrifuge at 3500 rpm for 10 min. The dashed lines represent the initial oil levels of the dispersions.

The so-called gel volume test is often used to evaluate the gel strength, measuring the sediment level at a given time at rest or centrifugation speed.^38^Figure 4 shows such data for the 5 wt % dispersions, containing *c*_H_2_O_ = 0.25 wt % that were prepared at 0, 45, 65, and 85 °C homogenization temperatures, and centrifuged at 3500 rpm for 10 min. Clearly, the gel volume increased with the homogenization temperatures (according to protocol B). At 85 °C (purple), the gel volume is about 60% of the original volume and 5 times less (∼12%) than that for the dispersion prepared at 0 °C. This is in agreement with the increase of the elastic modulus observed in the rheological measurements with homogenization temperature (Figure 3B). We expect the strength of colloidal gels or the resistance to sedimentation to be mostly controlled by the number and strength of the bonds between particles in the organoclay gel network. Therefore, increased gel strength would mean an increased number of contacts and/or increased bond strength. An increase in the number of contacts at a constant nominal clay concentration can be achieved by further exfoliation of the organophilic clay, as this would lead to an increase in the number density of primary particles and therefore the number of contacts. Such a scenario of temperature-induced promotion of further exfoliation was investigated using X-ray scattering, as discussed below.

### 3.3 X-ray Scattering To Evaluate Exfoliation with Homogenization Temperature

**Figure 5:**
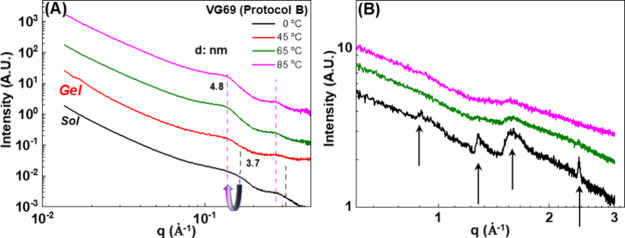
(A) SAXS and (B) WAXS scattering Intensity vs scattering wave vector *q* of VG69 dispersions (*c* = 5 wt %) prepared according to protocol B at 0 (black), 45 (red), 65 (green), and 85 °C (magenta). All the measurements were performed at 25 °C at a high clay concentration after centrifuging the dispersions at 3500 rpm for 10 min as described in Figure 4 with their corresponding concentration.

Aggregates like stacks (face-to-face) and tactoids (side-to-side) are typical of clay particles, and they are difficult to completely avoid; therefore, it should be expected that they are also present in the organoclay powder used here to prepare our suspensions. Under certain conditions, these should exfoliate further during the dispersion procedure. The level of exfoliation is typically characterized by X-ray scattering measured through WAXS (wide-angle X-ray scattering) and SAXS (small-angle X-ray scattering) probing the crystallinity of the clay particles and the distance between the clay planes giving rise to a basal peak. Swelling through the intercalation of solvent or surfactant between layers leads to a shift of the basal peak that can be monitored through X-ray scattering. Figure 5 shows such SAXS and WAXS scattering intensity *I*(*q*) data for the dispersions shown in Figure 4. A small amount of the centrifuged gels was placed in a washer holder placed in the X-ray beam.

SAXS spectra (Figure 5A) reveal a change in the peaks in the 0.13–0.17 Å^–1^ and in the 0.26–0.34 Å^–1^ range as the homogenization temperature increases. In this range, we expect contributions from first-order (001) and second-order (002) basal peaks arising from the interlayer spacing in clay stacks. The d values (derived from ) are 3.7 nm (0.17 Å^–1^) and 4.8 nm (0.13 Å^–1^), which are much larger than the 1 nm values observed in bentonite. We attributed this to the intercalation of surfactants between the clay layers.^36,62,65−67^ We interpret the evolution with homogenization temperature as arising from the decrease of the 3.7 nm contribution (first order 0.17 Å^–1^ and second order 0.34 Å^–1^) compared to the 4.8 nm contribution (first order 0.13 s order 0.26). We attribute this to a temperature and homogenization-induced swelling and exfoliation of the organo-bentonite stacks with the 3.7 nm spacing. However, contribution with 4.8 nm remain at all temperatures. At a larger wave vector (WAXS Figure 4b), the typical feature of crystalline bentonite is clearly seen for dispersions prepared at 0 °C and much less so at higher homogenization temperatures. We attribute this evolution to the presence of crystal-like particulate blocks in the original powder that remain under the 0 °C preparation conditions but dissolve further into smaller ones during homogenization at higher temperatures. Our measurements are in agreement with the literature where such spectra have been attributed to an almost complete exfoliation of primary particles (*N* > 2) and the formation of tactoid structures.^33,56,68,69^

### 3.4 Plateau Modulus of Stable Gels, *G*_P_ vs *c*_clay_

**Figure 6:**
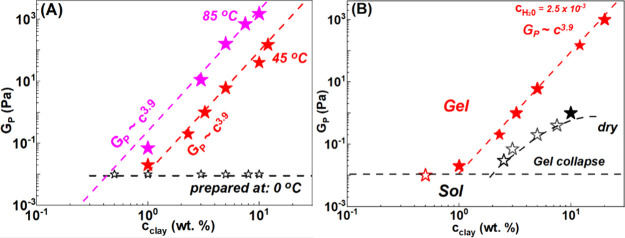
Evolution of plateau modulus (*G*_P_) as a function of clay concentration measured at ω = 1 rad/s and γ = 0.1% for VG69 dispersions prepared at (A) 0 °C (black stars), 45 °C (red stars) and 85 °C (magenta stars) at *c*_H_2_O_ = 0.25 wt % and (B) 45 °C at *c*_H_2_O_ = 0.25 wt % (red stars) and dry (black stars) conditions. Red stars in (B) were derived from measurements in ref (29) and are in agreement with the new measurements shown here in (A).

Establishing the influence of the particle concentration on the rheological properties of the colloidal gels, such as the plateau modulus and the yield stress, is an essential part of the colloidal gel characterization. The concentration dependence is often used as a way to identify the type of gel network formed. In particular, the power law dependence is discussed in terms of fractal networks and models have been proposed to link the power law increase of the elastic modulus to the structural fractal dimension.^70,71^ The dependence of the plateau modulus with organoclay concentration is shown in a log–log representation in Figure 6 for different preparation conditions, homogenization temperatures, and *c*_H_2_O_. Figure 6A shows the evolution of *G*_P_ measured at 25 °C with organoclay concentration prepared at different homogenization temperatures 0 (open black), 45 (red stars), and 85 °C (magenta stars). As already discussed, dispersions prepared at 0 °C always undergo fast sedimentation and do not develop elasticity (i.e., a solid-like behavior). At the same clay concentration, dispersions prepared at higher temperatures form gels. The scaling of *G*_P_ with *c*_clay_ is practically the same for dispersions prepared at different temperatures and follows a power law increase as *G*_P_ ∼ *c*_clay_^3.9^.

The strong evolution in *G*_P_ with *c*_clay_ is typical of colloidal gels where the attraction strength is large enough to form self-sustaining networks.^29^ The scaling exponent *G*_P_ ∼ *c*_clay_^3.9^ is similar to those reported for many other colloidal gels at low-volume fractions attributed to the fractal character of the network and its corresponding fractal dimension.^29,72−77^ Hence, we hypothesize that the presence of water molecules connects the building blocks into a network. The usual explanation in such cases is the H-bonding mediated attraction. Too low an amount of water precludes the formation of the self-supporting networks.^29^

In Figure 6B, we report the *G*_P_ vs *c*_clay_ of the dry prepared dispersions (black stars) and compare them with the standard homogenization conditions (with no thermal bath used and homogenization temperature reaching 45 °C with *c*_H_2_O_ = 0.25 wt %) (red stars). The dry condition preparation does not lead to stable gels as can be seen in Figure S2. We nonetheless report the low storage modulus values measured at ω = 1 rad/s, for comparison reasons. Only at very high clay concentration, *c*_clay_ = 10 wt %, we observe the formation of a weak gel (*G*_P_ = 1 Pa), which remained 2 orders of magnitude weaker than the same 10% dispersion but with *c*_H_2_O_ = 0.25 wt % (*G*_P_ = 100 Pa). We speculate that this weak gel formed at a high concentration of the dried particles is of a different type of solid-like metastable state than the attractive gels formed in the presence of water, likely a repulsive glass.

### 3.5 *G*_P_ Evolution with *c*_H_2_O_

**Figure 7:**
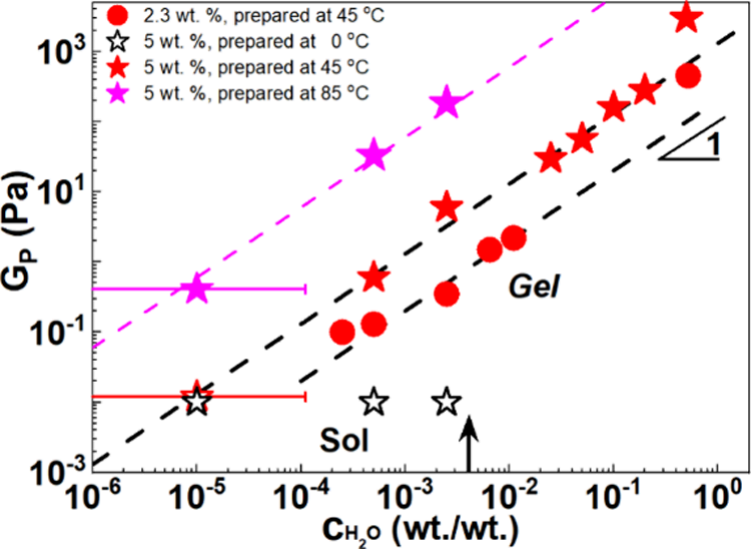
Evolution of plateau modulus (*G*_P_) with *c*_H_2_O_ (measured at ω = 1 rad/s and γ = 0.1%, 25 °C for VG69 dispersions at 2.3 (circles) and 5 wt % (stars) prepared according to protocol C at 0 (black), 45 (open red), and 85 °C (magenta symbols). The black arrow on the *x*-axis indicates the water in oil solubility limit; thus, below this concentration we expect molecularly dissolved water molecules whereas above we expect the formation of aqueous droplets. The transition point is expected to move to higher concentrations in the presence of clay due to the ability of clay to accommodate adsorbed water molecules. Filled symbols indicate a gel state and open symbols an unstable sol (liquid) state.

We now report and discuss the effect of increasing the water amount on the gel elastic modulus. Figure 7 shows the evolution of the plateau modulus, *G*_P_ with *c*_H_2_O_ for organoclay dispersions (2.3 and 5 wt %, circles and stars, respectively) (Protocol Β) at 0 (black), 45 (red), and 85 °C (magenta). Strikingly, the modulus of the gelled dispersions increases linearly with the water concentration, for all the different preparation conditions and clay concentrations. The effect of homogenization temperature is such that the curves are shifted upward to higher *G*_P_ values essentially retaining their linear increase with water content. For very high *c*_H_2_O_ (50 wt %) a deviation from linearity with a steep increase in the elasticity was observed. It should be noted that for such large water contents (*c*_H_2_O_ = 50 wt %). The gels are stable at rest but destabilize, phase-separate, and sediment upon weak agitation.

The linear evolution of *G*_P_ with *c*_H_2_O_ was also observed in dispersions with lower clay concentration *c*_clay_ = 2.3 wt % (red circles). This is the clay concentration used in a parent system relevant for drilling fluids studied before.^19^ The complete dynamic frequency sweep data of organoclay dispersions at *c* = 2.3 wt % (following protocol B at 45 and 85 °C), are shown in Figure S3.

As we do not expect any increase in clay exfoliation upon increasing the water content, the modulus increase cannot be ascribed to an increase in the total effective clay concentration. We rather attribute such an increase to a strengthening of interparticle attractions and possibly an additional network rearrangement that may lead to an increase in the average number of contacts.

As for how water mediates the interparticle contacts, we presume there should be at least two mechanisms. At a low water content regime, water is present in the form of molecules adsorbed on the clay surface forming H-bond bridges between clay particles, probably between the silanol groups at the edges of the clay particles.^78−80^ Once all adsorption sites have been covered, water molecules should start to form multilayers and eventually liquid-like water domains. Based on the surface/volume of clay particles, water adsorption on the clay surfaces amounts to a maximum of a few % water as discussed in the TGA section above. The solubility of water in oil is also of the order of a few wt %. Therefore, above a few % of water content in the dispersion, water should be present in liquid droplet form. Liquid water between particles may then lead to capillary bridges that would provide effective attraction between particles as in capillary gels.^54,81^ We note that the presence of a stabilizing surfactant for the organoclay may provide a way to form reverse micelles of water in oil. Interestingly the expected regimes where the two mechanisms would dominate are not seen in the evolution *G*_P_ with *c*_H_2_O_ in Figure 7. The concentration dependence is still linear over a broad range of *c*_H_2_O_ concentrations, far above the one expected for the saturation of water adsorption and the formation of water droplets. We further recall that the presence of H_2_O is a necessary condition for stable gels but not a sufficient one, as they also require a large enough homogenization temperature and rate.

### 3.6 Yield Stress and High Shear Viscosity of Gels

**Figure 8:**
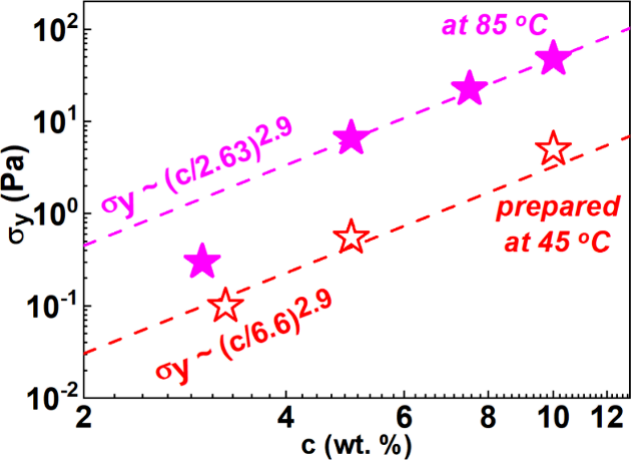
Yield stress (σ_y_) as a function of clay concentration for VG69 dispersions prepared according to protocol C, at 45 °C (open red stars) and 85 °C (magenta stars) at *c*_H_2_O_ = 0.25 wt %.

We next report the flow behavior of different gels prepared under varying conditions. As these are yield stress samples, we focus on their yield stress (σ_y_) and the infinite-shear fluid viscosity (η_∞_), two quantities defining how such samples flow under the application of shear stress. Both are deduced from a steady shear rate sweep (flow curve) measured at 25 °C first with increasing shear rate and subsequently upon decreasing shear rate. Typical steady flow curves for organoclay dispersions prepared under different conditions are shown in Figure S4. We note that several flow curves exhibited an additional stress decrease at the low shear rate regime stress plateau, typical of wall slip, that is common in colloidal gel systems,^82^ this part of the flow curve was not considered in the analysis. The dynamic yield stress was identified as the low shear rate limit stress using the Spicer model^83^, where γ̇_c_ is the critical shear rate attributed to plastic contributions and η_bg_ is the background viscosity. Figure 8A shows the evolution of σ_y_ with organoclay concentration for preparation temperatures 45 and 85 °C. σ_y_ increases sharply with clay concentration for both homogenization temperatures following a power law dependence, σ_y_ ∼ *c*_clay_^2.9^, with the absolute values significantly higher (15 times) for the dispersions prepared at 85 °C as can be seen in Figure 8A.

On the high shear rate side, we did not reach the limiting high shear rate viscosity (which is independent of the shear rate). We therefore report, in Figure S4, the value of the viscosity at the maximum measurable shear rate, at γ̇ = 1000 s^–1^. It appears to have different scaling with organoclay concentration with η_1000_ ∼ *c*_clay_^1.3^ and η_1000_ ∼ *c*_clay_^1.9^ for 45 and 85 °C homogenization temperatures, respectively.

**Figure 9:**
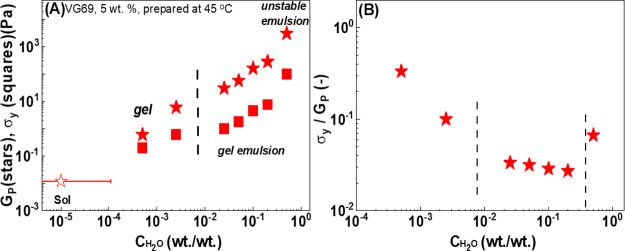
Evolution of (A) plateau modulus (*G*_P_) and yield stress (σ_y_) and (B) ratio of yield stress over the plateau modulus  of VG69 (5 wt %) dispersions prepared according to protocol C at different *c*_H_2_O_. All of the measurements were performed at 25 °C. The vertical lines indicate different water concertation regimes, as discussed in the text. The dashed line at a low water concentration indicates the water to oil solubility limit. The dashed line at a higher water concentration indicates the transition from an emulsion gel to an unstable emulsion.

In Figure 9A, we report σ_y_ with increasing *c*_H_2_O_ for dispersions prepared at 45 °C and compare it with the corresponding dependence of *G*_P_. The original flow curves of the dispersions for different *c*_H_2_O_ concentrations can be found in Figure S5. Despite the limited number of data points, the yield stress seems to exhibit a concentration dependence different from that of the plateau modulus, *G*_P_, although both increase monotonically with water content, with the increase of *G*_P_ being essentially linear.

For both the yield stress and the elastic modulus, the highest water content (50 wt %) deviates from the linear increase. However, we should note that the flow curve at this high-water content shows a strong indication of shear-induced phase separation that is the underlying trigger of wall slip, as shown in Figure S5B, so the value of the extracted yield stress should be considered with caution.

In Figure 9B we report the ratio of yield stress over the plateau modulus, which defines a measure of the yield strain, . The data suggest the existence of three regimes, indicated by vertical dashed lines. At low water content, lower than *c*_H_2_O_ < 1 wt % this effective yield strain decreases with increasing water amount, indicating that the sample becomes increasingly more fragile. At intermediate water content, between 1 wt % ≤ *c*_H_2_O_ ≤ 20 wt %, the effective yield strain is lower, around 0.03, and weakly decreases as water content increases. At higher water content (*c*_H_2_O_ = 50 wt %), the data indicate an increase in the yield strain.

### 3.7 Rheological State Diagram and Gelation Scenario

**Figure 10:**
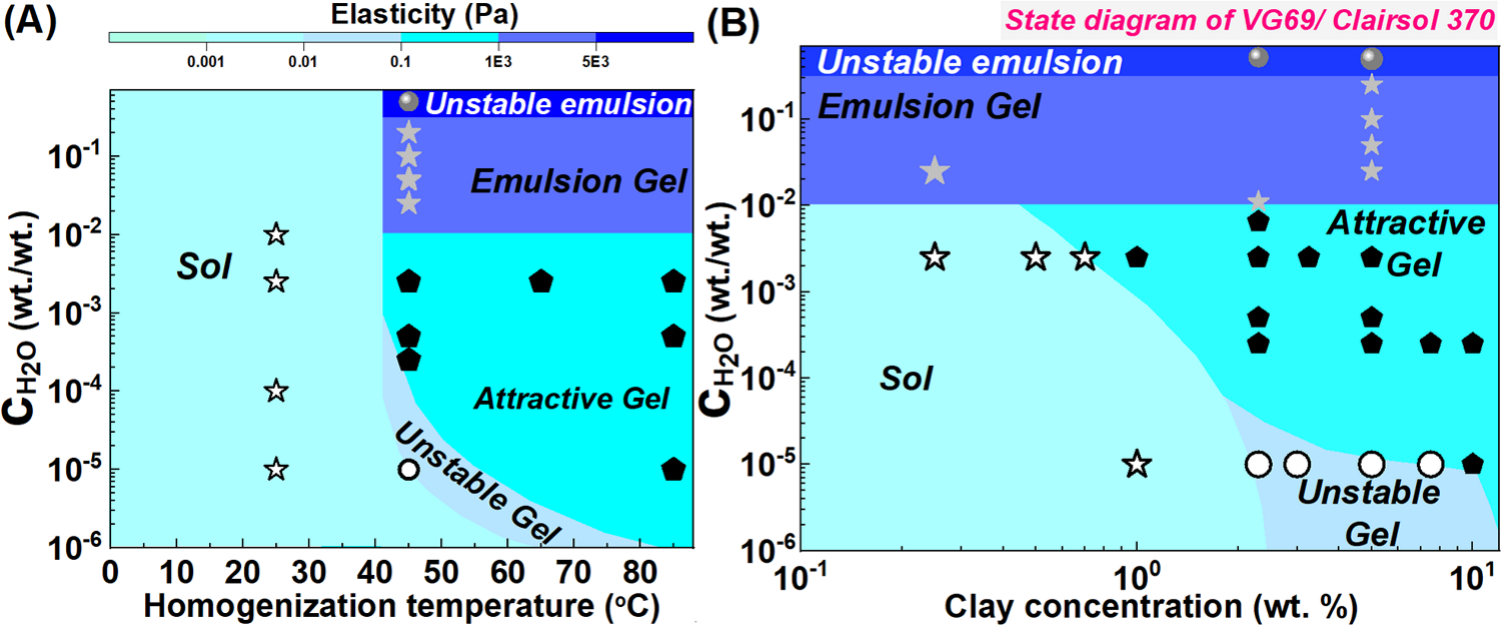
(A) Dynamic state diagram of VG69/Clairsol 370 (5 wt %) dispersions at 25 °C in preparation temperature—*c*_H_2_O_ space. Border lines between different regimes are approximate based on the available systems studied. Different colors indicate dispersions with different elasticity, as indicated in the scale bar at the top. (B) State diagram of VG69/Clairsol 370 dispersions at 25 °C in *c*_H_2_O_—clay concentration space for dispersions prepared at 45 °C. Open stars attributed to sols (with fast sedimentation), open circles to unstable gels (with slow gel collapse), filled pentagons to stable attractive gels, filled gray stars to emulsion gels, and gray sphere to unstable emulsions (showing phase separation upon shear).

We now summarize our observations of the organoclay dispersions (*c* = 5 wt %) rheological state at 25 °C for the different preparation conditions in state diagrams with *c*_clay_ or temperature on the horizontal axis and *c*_H_2_O_ on the vertical axis. These are shown in Figure 10A,B using color coding that reflects the shear storage modulus. Low homogenization temperature leads to unstable sols independent of the added water amount (*T* < 40 °C). At larger homogenization temperatures, unstable collapsing gels are observed at low water content. Reaching a large water amount, the formation of water in oil emulsions is expected due to the limited solubility of water in oil (estimated at *c*_H_2_O_ = 0.5 wt %) and in organoclay. The onset of droplets formation can be detected through a change of optical characteristics of the dispersions as strong scattering by the droplets leading to a more turbid and “creamy” image. We have (arbitrarily) set the emulsion domain above 1% water, as shown in Figure 10. We further distinguished stable gel emulsions at intermediate water content (*c*_H_2_O_ ≥ 1 wt %) and unstable gel emulsions at higher (*c*_H_2_O_ ≥ 30 wt %) (see also Figure 9).

We conjecture that during the homogenization stage, swelling in combination with breaking and exfoliation of aggregates present in the powder causes an increase in the effective organoclay volume fraction and an increase in the number of interparticle contacts. This is in part confirmed by the SAXS-WAXS data. As a result, *G*_P_ and σ_y_ increase, in a way similar to their increase with clay concentration (at constant homogenization temperature) (Figure 6, 8). The observed *G*_P_ increase between the 45 and 85 °C homogenization temperature (Figure 7) by a factor of 15 may thus be caused by an increase in the effective clay volume fraction by a factor of 2.

**Figure 11:**
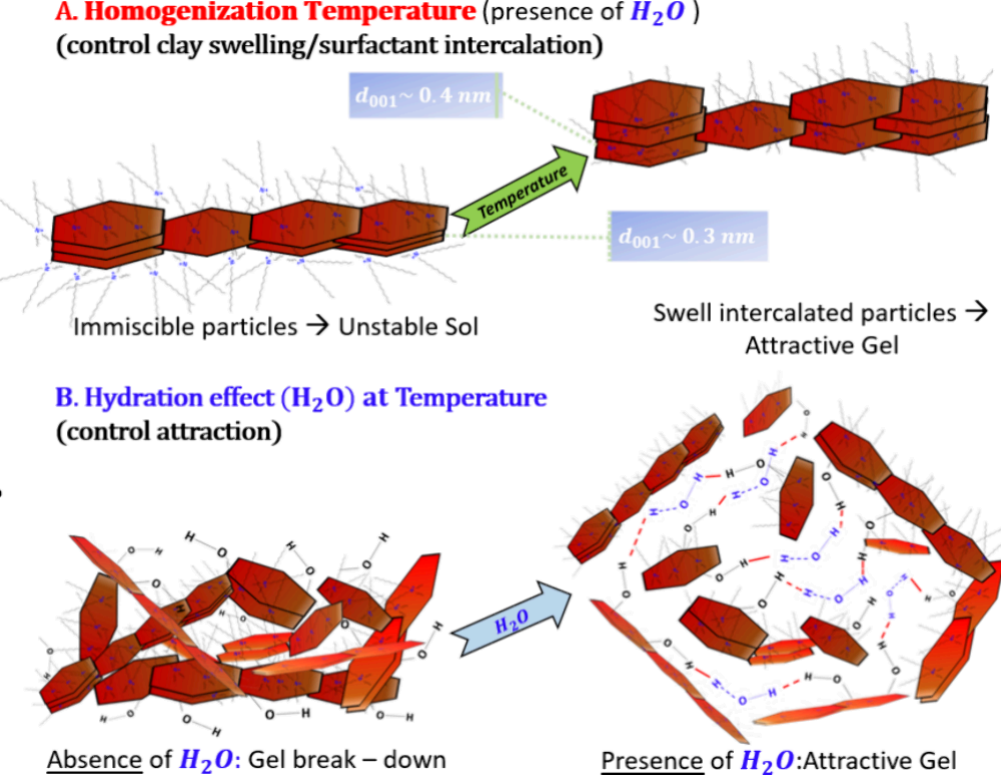
Tentative sketch of particle microstructure in VG69 dispersions prepared at different preparation protocols. (A) shows the effect of homogenization temperature (in the presence of H_2_O) on the tactoid aggregate; low homogenization temperature (left top) leads to dispersion with immiscible particles (unstable sol state), whereas high homogenization temperature (right top) leads to swell intercalated particles (attractive gel state). (B) Hydration effect for dispersions prepared at *T* ≥ 45 °C, in dry conditions (bottom left) the tactoids undergo precipitation and the dispersion is in unstable sol or in gel breakdown state, while in wet conditions (bottom right), the tactoids are stabilized by the H-bond and form an attractive gel.

The rheological data suggest that *c*_H_2_O_ primarily affects the strength of the interparticle attractions. Dry organoclay dispersions are not capable of developing a gel state and after a short waiting time (typically minutes) the gel collapses and sedimentation takes place. This inability to form stable gels in dry conditions highlights the importance of water molecules in the stability of the gels (Figure 10). When water is added during the homogenization stage, the formation of strong gels is observed. We attribute the formation of gels to the onset of strong enough interparticle attractions upon the addition of water. Interestingly, elasticity seems to increase linearly with the water amount (*G*_P_ ∼ *c*_H_2_O_) up to a rather large water concentration. The mechanisms controlling the colloidal gel stability are generally discussed in terms of attractive forces between clay particles. In the case of organoclays in oil, two types are expected to come into play, water bridges between particles through H-bond connection with silanol groups present at the clay surface and van der Waals interaction between clay particles, not fully screened by the adsorbed surfactant. The sketch in Figure 11 illustrates our understanding of the effect of homogenization temperature and water content during preparation: water adsorbs on the organoclay, enabling the ability to form bridges and allow the formation of interconnected clay particles. Moreover, further exfoliation and aggregate breaking are achieved especially at higher homogenization temperatures. Although a detailed understanding of the molecular mechanisms involved is still not reached it is clear that water molecules are required to “switch-on” attraction and lead to stable (nonsedimenting) gels.

At high water content (above the solubility limit in both organoclay and mineral oil), mesoscopic and eventually macroscopic water droplets form. This impacts the optical conditions of the dispersion adopting the typical emulsion look. The systems remain a stable gel, which, however, we now denote as an emulsion gel. In this regime, the formation of capillary bridges is probable, and their effect on the rheological properties of the specific system would need further investigation. Eventually, when water exceeds 30 wt %, the gels become mechanically unstable, as a mechanical disturbance induces the separation between oil-rich and water-rich phases.

At this point, we want to compare the 50% water with a parent system that was previously introduced as a model for drilling fluids (MDF) and studied.^19^ Both contain the same organoclay/oil/water proportions, but the MDF includes extra cosurfactants, while brine is used instead of pure water. Both systems have an elastic response with a measurable yield stress. However, the MDF showed a much different elastic modulus, *G*_p_ = 7 Pa compared to the values measured here (*G*_p_ = 420 Pa, see Figure 7) for the 50% water sample. Moreover, the MDF was stable, unlike the 50% water that tends to demix. Both systems are water-in-oil emulsions, as visible in the microscopy pictures shown in Figure S6 with some noticeable differences in droplet size and size distribution with the MDF showing larger and more monodisperse droplets. The large differences in both the modulus and yield stress indicate the important effect of the extra components (brine and extra surfactants) on the rheological properties. Part of our initial motivation for the present work was to reveal the role of the organoclay networks in the drilling fluid rheology; it is clear that extra components also have an influence; the organoclay in oil “activated” by water is not the only contributing factor in the rheology. A possible explanation would be that the extra surfactants further stabilize the emulsion while the CaCl_2_ brine affects the interclay attractions.

One of the striking findings of this study is the proportionality between water amount and the elastic modulus over a large range of water content. It would thus be interesting to check the possible generality in other organoclay systems. Nonetheless, it is obvious that the water content offers a simple way to control interactions and gel strength, which is much looked for in colloidal science (an ability to simply tune the effective attraction).

## 4 Conclusions

We have used shear rheology to identify the effect of preparation conditions on the rheological properties of dispersions of a commercial organo-bentonite. We showed how both the homogenization temperature and the water content affect the dispersion properties. Stable gels require a combination of high enough homogenization temperature (*T* > 0 °C), strong shear, and a minimal amount of water (at least 100 ppm). In the absence of added water, fast sedimentation is observed independent of the preparation temperature. Similarly, gels prepared at a lower temperature of 0 °C always showed fast sedimentation independent of the water amount and shearing conditions. Strikingly the elastic modulus of the gelled dispersions was found to vary linearly with water amount over a broad range of water concentrations ranging from 100 ppm to more than 10%. The water amount also influences the flow behavior and the gel fragility as the apparent yield strain decreases with increasing water amount. We established a state diagram, and we proposed a simple rationalization for it taking into account two distinct effects: that of temperature which promotes exfoliation and that of water addition which enhances interparticle attraction. Larger exfoliation levels promoted by temperature result in an increased number (and concentration) of building blocks, while the addition of water results in increased “bridge” interaction between building blocks. The gelled dispersions showed high stability over long periods of time (months instead of minutes or hours for the untreated dispersions).

At water content over a few wt %, the formation of water droplets is observed as expected from the oil solubility value. The dispersion becomes an emulsion upon homogenization. Dispersions remained stable up to 30 wt % water in oil, and larger water content led to dispersion demixing into water-rich droplets in an oil-rich continuous phase upon shear.

Our findings give clear insights on how we can control the rheological properties of organoclay dispersions through temperature and preparation protocols.

## Supporting Information Available

SEM images of organoclay particles; linear viscoelastic spectra of organoclay dispersions prepared according to different protocols; flow curves and high shear viscosity of organoclay dispersions prepared at 45 and 85 °C and different water contents; and microscopy images of organoclay dispersions and a stabilized emulsion with clay (PDF)
