# Supplementary material for: How Preparation Protocols Control the Rheology of Organoclay Gels
Source: Ind Eng Chem Res. 2025 Mar 22;64(13):6980–91. doi: 10.1021/acs.iecr.4c04467 (PMC11970213; doi:10.1021/acs.iecr.4c04467)
Supplement: Supplementary file 1 — ie4c04467_si_001.pdf [file ie4c04467_si_001.pdf]

Supporting Information

How preparation protocols control the rheology of organoclay gels

Nikolaos A. Burger[1,2], Benoit Loppinet[1], Andrew Clarke[3] and George Petekidis[1 2]*

[1] IESL-FORTH, Vassilika Vouton, Heraklion, 70013 Greece
[2]Department of Materials Science & Engineering, University of Crete, Heraklion 70013, Greece
[3]SLB, Schlumberger Cambridge Research, High Cross, Madingley Road, Cambridge CB3 0EL, UK

*Email: burger_nik0s@hotmail.gr

Organoclay characterization. VG69 organoclay consist of polydisperse plates. More details on the chemical composition of the clay exist in [1].

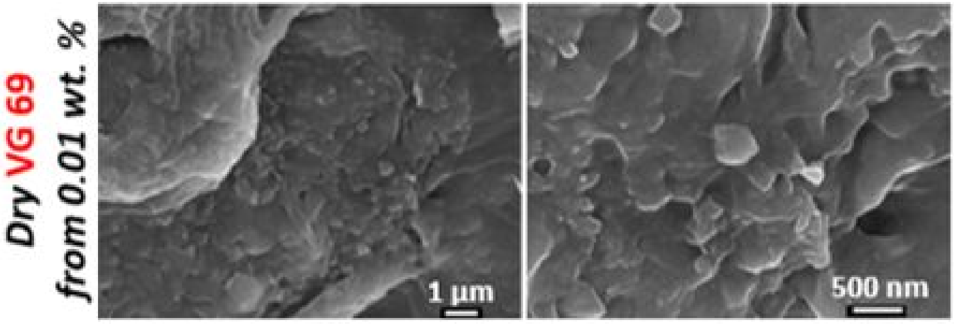

Figure S1. SEM images of VG 69 platelets dry powders at different magnifications where scale bars indicate 1 µm (left) and 500 nm (right). Images taken after drying $0.01 wt.\%$ clay- heptane dilute dispersions.

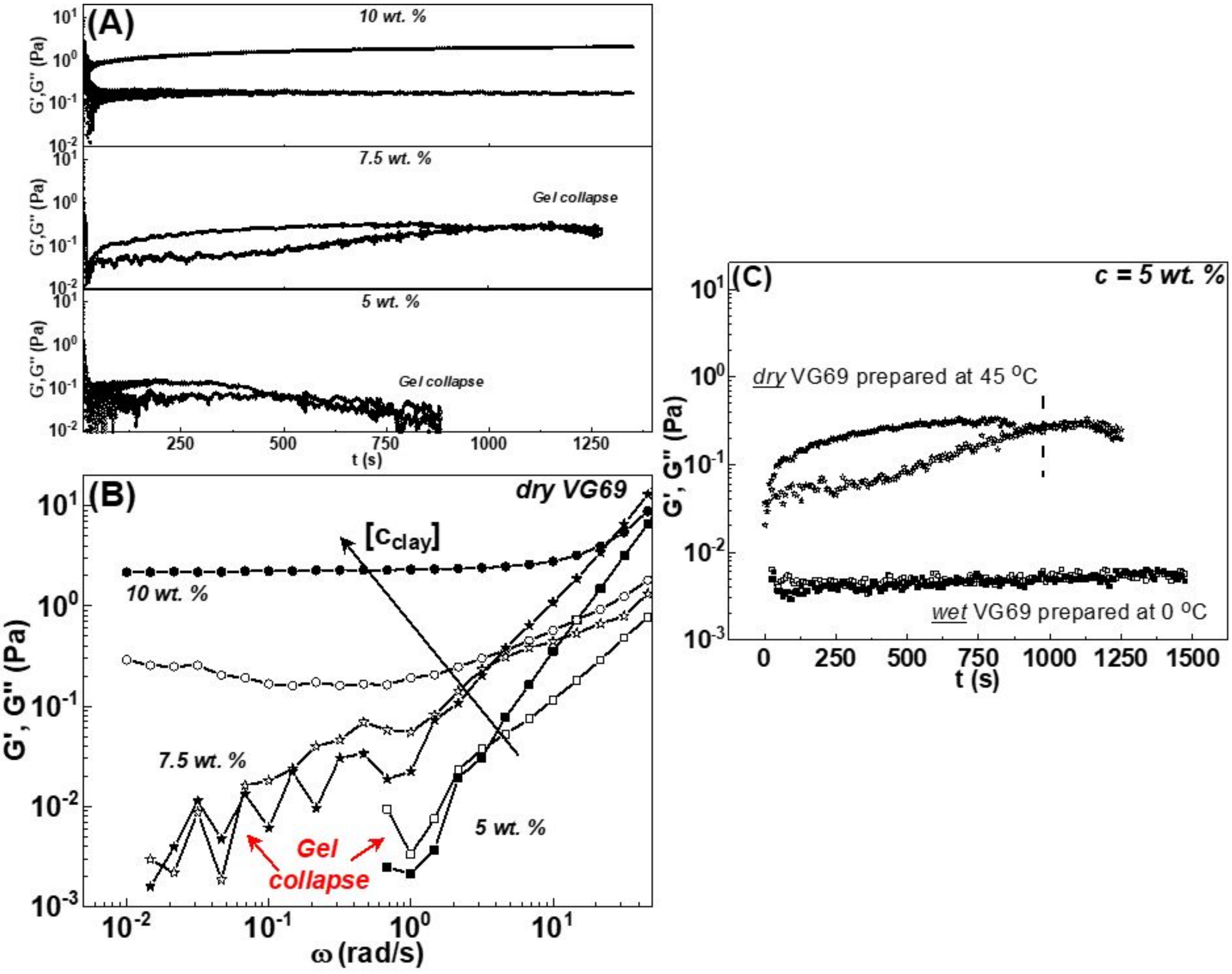

Figure S2. Storage (G', full symbols) and loss (G'', open symbols) modulus (Pa) as a function of: (A) waiting time (in seconds) and (B) frequency at different dry VG69 concentrations (5, 7.5 and 10 $wt.$%) measured at $\gamma = 0.1$ at $\omega = 1 rad/s$ and (C) waiting time for dispersions (5 $wt.$%) prepared according to protocol B (black squares) and C (black stars).

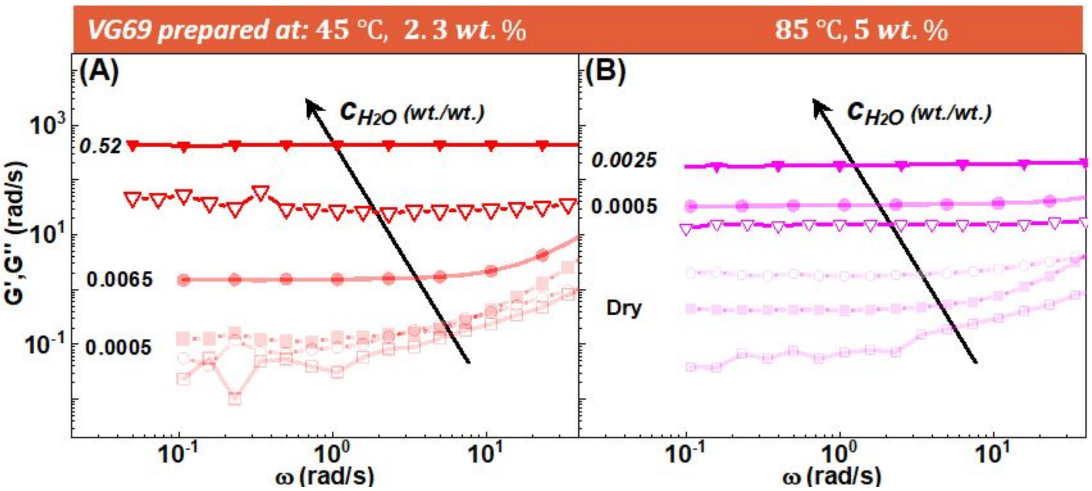

Figure S3. Storage (G', filled) and loss (G'', open symbols) modulus (Pa) as a function of frequency of VG69 dispersions prepared according to protocol B at (A) 45 ℃, 2.3 $wt.$% and (B) 85 ℃, 5 $wt.$%.

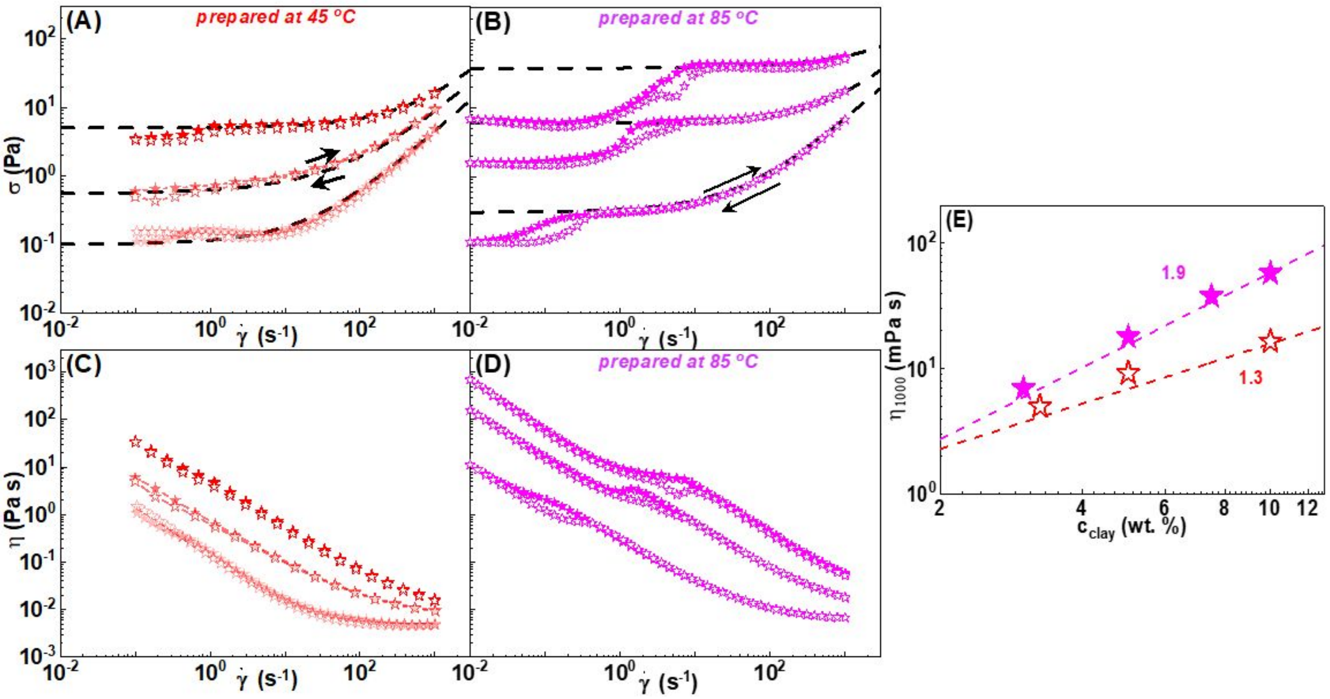

Figure S4. (A) Evolution of σ from high to low (filled) and low to high (open symbols) shear rates for VG69 dispersions prepared according to protocol B at (A) 45 ℃ (3.25, 5 and 10 wt.%) and (B) 85 ℃ (3, 5 and 10 wt.%). Respective shear viscosities depicted in (C) and (D). All the measurements performed at 25 ℃. For the evaluation of yield stress, we used the Spicer model $\sigma = \sigma_y + \sigma_y(\frac{\dot{\gamma}}{\dot{\gamma}_c})^{0.5} + \eta_{bg}\dot{\gamma}$ where $\dot{\gamma}_c$ is the critical shear rate attributed to plastic contributions and η_{bg} is the background viscosity.

(E) high shear viscosity, η_{1000} measured at $\dot{\gamma} = 1000\ \text{s}^{-1}$ as a function of clay concentration for dispersions prepared at 45 (open red stars) and 85 (filled magenta stars) ℃, respectively.

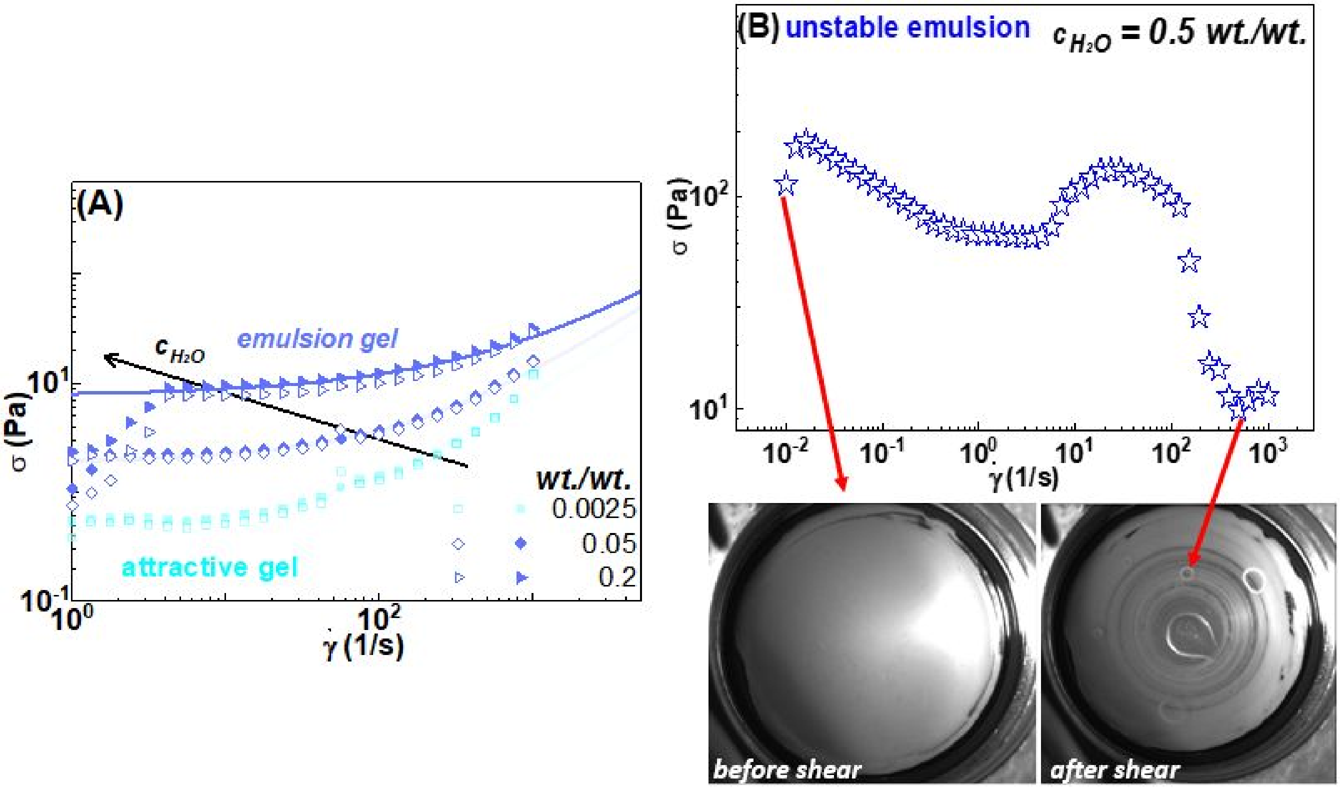

Figure S5. (A) Evolution of σ from high to low (filled) and low to high (open symbols) shear rates for VG69 (5 wt.%) dispersions prepared according to protocol (c) at 45 ℃ for different c_{H_2O}. (B) Evolution of σ from low to high shear rates for VG69 (5 wt.%) dispersions prepared according to protocol C at c_{H_2O} = 50 wt.% Images extracted at rest through white light illumination low magnification imaging before (left) and after (right) shear rate treatment. The data from different phases, i.e., an attractive gel, emulsion gel (A) and unstable emulsion (B) are denoted by different colors. All the measurements were taken at 25 ℃.

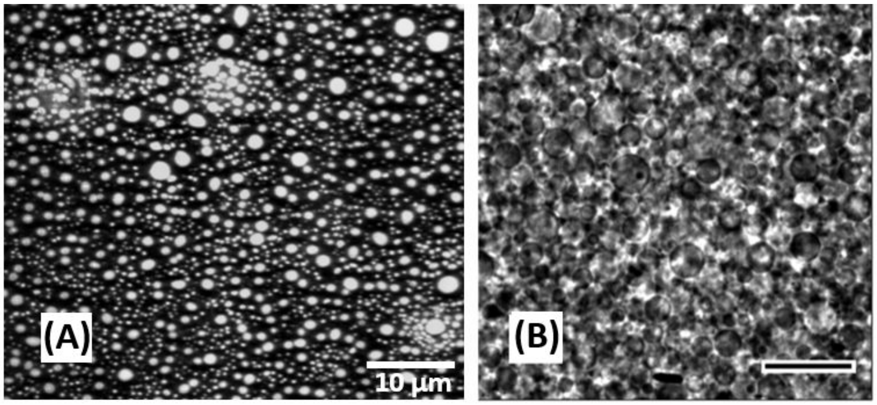

Figure S6. Comparison of microstructure of (A) unstable emulsion with clay (2.3 wt.% clay dispersion) in 50 wt.% water in oil derived from optical microscopy in reflection mode, as in Figure 3 and (B) stabilized emulsion with clay, (VV1) derived from optical microscopy in transmission, adapted from [2]. The scale bars in (A) and (B) indicate 10 μm scale.

(1) Bergane, C.; Hammadi, L. Impact of Organophilic Clay on Rheological Properties of Gasoil-Based Drilling Muds. *J. Pet. Explor. Prod. Technol.* **2020**, *10* (8), 3533–3540. https://doi.org/10.1007/s13202-020-01008-x.

(2) Clarke, A.; Jamie, E.; Burger, N. A.; Loppinet, B.; Petekidis, G. A Microstructural Investigation of an Industrial Attractive Gel at Pressure and Temperature. *Soft Matter* **2022**, *18* (20), 3941–3954. https://doi.org/10.1039/D2SM00248E.
